# Who Am I? Eyebrow Follicles Minimize Donor-Derived DNA for Germline Testing After Hematopoietic Stem Cell Transplantation

**DOI:** 10.3390/ijms27020744

**Published:** 2026-01-12

**Authors:** Matthias Mertens, Mona Sadlo, Jörn-Sven Kühl, Klaus Metzeler, Louisa Zschenderlein, Jeanett Edelmann, Claudia Lehmann, Sarah Thull, Mert Karakaya, Clara Velmans, Theresa Tumewu, Matthias Böhme, Christina Klötzer, Anne Weigert, Vladan Vucinic, Julia Hentschel, Mareike Mertens

**Affiliations:** 1 MIT Future Tech, MIT Sloan School of Business, Massachusetts Institute of Technology, Cambridge, MA 02139, USA; 2 Institute of Human Genetics, University Medical Center Leipzig, 04103 Leipzig, Germany; 3 Department of Pediatric Oncology, Hematology and Hemostaseology, University of Leipzig, 04103 Leipzig, Germany; 4 Department of Hematology, Cellular Therapy and Hemostaseology, Leipzig University Hospital, 04103 Leipzig, Germany; 5 Leipzig Medical Center, Institute of Legal Medicine, Leipzig University, 04103 Leipzig, Germany; 6 Institute for Transfusion Medicine, University Hospital Leipzig, 04103 Leipzig, Germany; 7 Institute of Human Genetics, Faculty of Medicine and University Hospital, University of Cologne, 50937 Cologne, Germany; 8 Institute of Human Genetics, University Hospital Düsseldorf, Heinrich-Heine-University, 40225 Düsseldorf, Germany; 9 Division of Genetics and Genomics, Boston Children’s Hospital, Harvard Medical School, Boston, MA 02115, USA

**Keywords:** germline diagnostics, chimerism, post-transplant diagnostics, tissue source

## Abstract

Germline genetic testing plays a critical role in diagnosing inherited predispositions and increasingly guides therapeutic and surveillance choices—but becomes technically challenging after allogeneic hematopoietic stem cell transplantation (HSCT), when donor-derived DNA contaminates host tissues. To address this, we compared donor-derived DNA across three accessible tissues—buccal swab, nail, and eyebrow follicles—in recipients after hematopoietic stem cell transplantation using two orthogonal assays (34-SNP next-generation sequencing and a 27-marker short tandem repeat panel) and modeled clinical covariates that influence chimerism. Eyebrow follicles showed consistently low donor DNA (median 1% by NGS; 3% by STR) whereas buccal swabs and nails carried substantially higher donor fractions (+25 and +22 percentage points versus eyebrow, respectively; both *p* < 0.01). Across methods, STR yielded on average ≈6 percentage points higher donor fractions than NGS at low-level chimerism. Several transplant covariates correlated with chimerism: matched-related donors and a perfect HLA match (10/10) were each associated with lower donor DNA (≈12–14 and 15–20 percentage points, respectively); longer times since hematopoietic stem cell transplantation correlated with lower levels for nail samples, and donor–recipient sex match correlated with higher donor DNA (~7–8 percentage points). Even low-level chimerism can distort germline variant interpretation. We propose a pragmatic protocol for post-hematopoietic stem cell transplantation germline testing that prioritizes eyebrow follicles as the default tissue. An SNP-based quality control assay is used to flag unsafe donor fractions (≥ 5–10%) before comprehensive germline analysis, reducing the risk that chimeric donor DNA distorts germline variant interpretation.

## 1. Introduction

Germline genetic results increasingly inform therapy, surveillance, cascade testing, and pharmacogenomics. After allogeneic HSCT, however, blood—the default substrate for genetic testing—becomes unreliable due to donor-derived DNA [1,2,3,4,5,6,7]. Whenever available, pre-HSCT peripheral blood remains the preferred germline reference; when unavailable, laboratories and clinics increasingly turn to non-blood tissues—chiefly buccal mucosa, nails, cultured skin fibroblasts, and hair follicles—to infer the recipient’s germline after HSCT, yet the reliability of these matrices remains unsettled [4,5,6,7,8,9,10,11,12,13,14,15,16]. 

Cultured skin fibroblasts are commonly used but may be impractical due to invasiveness, turnaround time, or patient condition—motivating evaluation of non-invasive alternatives such as hair follicles, nails, and buccal swabs [17,18]. The literature illustrates why clinicians receive mixed messages. On one hand, early observations suggested that hair (especially when sampling shafts rather than roots) could represent the recipient’s genotype with minimal donor admixture, supporting its adoption as a practical specimen [8]. On the other hand, subsequent series demonstrated detectable donor DNA in hair follicles after HSCT, consistent with reports of donor sequences in buccal swabs and nails [7,12,13]. These studies varied in sampling protocols, timing post-transplant, transplant characteristics, and—critically—in the assays used to measure admixtures. Although both short tandem repeat (STR) panels and next-generation sequencing (NGS)-based assays are used to assess chimerism, comparative evidence across tissues within the same patient, using orthogonal methods, remains limited. Furthermore, most prior studies were not designed to quantify how transplant-related covariates (e.g., HLA match, donor relatedness, sex match, time post-HSCT) influence extra-hematopoietic chimerism in everyday, clinic-collected samples. 

This knowledge gap has practical consequences. Even single-digit percentages of donor DNA can distort germline variant interpretation, particularly for heterozygous calls near the limit of detection, copy number inference, or variant allele fraction-based quality filters. False positives can trigger unnecessary cascade testing in families; false negatives can delay appropriate surveillance or therapy. To safeguard clinical validity, laboratories need (i) a default tissue with consistently minimal donor admixture, (ii) a brief QC step to recognize unsafe admixture before comprehensive analysis, and (iii) guidance on how patient and transplant characteristics should influence tissue choice and result interpretation.

Therefore, we performed a within-patient, cross-tissue study of buccal swabs, nails, and eyebrow follicles in HSCT recipients, applying both a 34-SNP NGS assay and a 27-marker STR panel to quantify donor-derived DNA and evaluate analytic performance. We then modeled clinical covariates associated with chimerism to identify scenarios in which certain tissues may be unsafe for germline inference. Our results support a default tissue choice and a minimal QC step that together reduce the risk of erroneous germline inference and enable confident integration of genomic medicine into post-transplant hematology care.

## 2. Results

### 2.1. Tissue-Specific Differences in Donor DNA Shares

**Figure 1:**
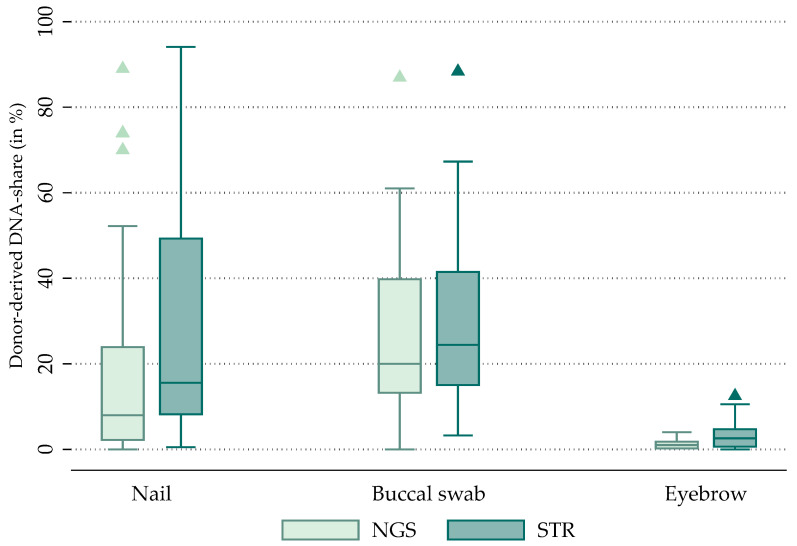
Differences in donor-derived DNA fractions across tissues. NGS: buccal n = 45, nail n = 45, eyebrow n = 28; STR: buccal n = 45, nail n = 44, eyebrow n = 28.

Figure 1 reports donor-derived DNA fractions for each analyzed tissue. The figure shows box plots of patient-specific median DNA shares for each tissue and method. See Table 1 for the associated statistics.

We find pronounced differences in donor-derived DNA fractions across the distinct tissue types. Notably, nail and buccal swab tissues exhibit high levels of donor-derived DNA with median values of 8% (NGS) and 16% (STR) for nails and 20% (NGS) and 24% (STR) for buccal swab tissues. Eyebrow hair follicle samples display considerably lower levels with median levels of 1% (NGS) and 3% (STR). 

### 2.2. Regression Analysis Results—Key Findings

Table 2 presents the results obtained from estimating various specifications of regression Equation (1) using ordinary least squares regression. Columns 1 to 5 display the results obtained when pooling NGS and STR datapoints. Columns 6 and 7 present separate regressions for STR and NGS data points. Using the STR assay yielded, on average, a 6-percentage-point higher donor-derived DNA fraction compared to NGS. This effect is statistically significant at the 1% level (column 1).

The most prominent result in our study pertains to tissue-specific differences. Eyebrow follicles show by far the lowest amount of donor-derived DNA. On average, buccal swab tissues exhibited a 25-percentage-point higher donor-derived DNA fraction than eyebrow follicle tissues. Similarly, nail tissues yield, on average, a 22-percentage-point higher donor-derived DNA fraction. These differences are consistently observed across both NGS and STR assays and are statistically significant at the 1% level (column 2). In column 3, we run the same regression as in column 2 but control for patient fixed effects, such that we compare differences in donor-derived DNA fractions across tissues within patients only. Our results are almost unchanged. As shown in column 4, incorporating control variables, such as DNA concentration and various cohort characteristics (e.g., age at transplantation to account for age-related factors), does not alter our primary findings. Importantly, the influence of DNA concentration on donor-derived DNA fractions is small and statistically insignificant. One notable result is that the coefficient for nail tissue changes markedly in column 5, where interaction terms are introduced. This reflects a change in interpretation: with interactions included, the main-effect coefficient no longer represents the unconditional average difference between nail and eyebrow tissues, but instead the conditional effect on the interacting variables being at their reference values. As discussed below, the interaction estimates indicate that donor-derived DNA levels in nail tissue are, on average, sensitive to time since transplantation, which explains the changes in the main effect. When analyzing NGS and STR data separately, our key results remain robust (columns 6 and 7). Notably, NGS results (column 7) remain similar after controlling for the total number of reads and average depth at the patient tissue level, which helps to alleviate concerns due to material depth discrepancies.

### 2.3. Regression Analysis Results—Other Clinically Relevant Results

Beyond tissue-specific disparities, our analysis in Table 2 reveals further novel insights. Donor and recipient sex matching correlated with approximately 7–8-percentage-point higher donor-derived DNA fractions (columns 4–7). A longer time since transplantation is associated with lower donor-derived DNA fractions in nail samples relative to eyebrow hair follicles (note: after inclusion of the interaction terms in column 5, the main effect of time since transplantation is no longer statistically significant, indicating that this main effect was driven by nail samples). A better HLA match is associated with substantially lower donor-derived DNA fractions (15–20 percentage points) in specifications without interactions, and this main effect even remains statistically significant when interactions are included, despite it declining in magnitude, possibly due to a large but statistically insignificant interaction effect between HLA match and nail tissue. Finally, we find strong evidence that HSCT from a matched related donor correlates with lower donor-derived DNA fractions of 12–14 percentage points, depending on the specification. Together with the results on the HLA match, this suggests that a better match quality has a strong negative impact on the donor-derived DNA fraction in the examined materials of the recipient. 

## 3. Discussion

This study provides quantitative, within-patient evidence that accessible non-blood tissues differ markedly in donor-derived DNA admixture after HSCT. Across three matrices, eyebrow follicles consistently showed the lowest donor DNA fractions (medians 1% by NGS; 3% by STR), whereas buccal swabs and nails exhibited substantially higher admixture (+25 and +22 percentage points relative to eyebrow, respectively). At low levels of chimerism, STR yielded on average ≈ 6 percentage points higher donor fractions than the NGS panel. While this difference is unlikely to alter classification of typical heterozygous germline variants (expected VAF ≈ 50%), it may affect borderline scenarios, such as distinguishing subtle allelic imbalance from true heterozygosity. Importantly, both assays converged on the same qualitative conclusion regarding tissue suitability, although laboratories should be aware that STR-based estimates may modestly overstate donor admixture at low levels. Since STR and NGS were performed on the same tissue DNA extracts, this systematic difference is most plausibly explained by analytical factors (e.g., marker composition and mixture/threshold behavior at low-level chimerism) rather than biological variation. Importantly, this offset did not change the clinical tissue suitability conclusion in our cohort (eyebrow follicles remained the preferred matrix across both methods).

Consistent with prior reports, buccal chimerism may be influenced by local inflammation or altered epithelial turnover. Thus, although our data indicate that buccal mucosa is generally more prone to donor admixture than eyebrow follicles, further research is needed to disentangle tissue-intrinsic susceptibility from modifiable collection- or health-related factors. This underscores the importance of empirically assessing tissue chimerism in individual clinical contexts before using buccal DNA for germline inference.

Since our cohort included both pediatric and adult recipients, age-related differences in epithelial turnover and immune reconstitution could plausibly influence extra-hematopoietic chimerism. In our multivariable model, age at transplantation showed at most a small association with donor-derived DNA fraction and did not alter our results. Nevertheless, validation in larger adult-only cohorts—especially older adults—will be important to confirm generalizability.

We also found that transplant-related covariates showed consistent associations across assays, model specifications, and patient fixed-effects analyses. A perfect HLA match (10/10) and matched related donors were associated with substantially lower donor admixture, with effect sizes of approximately 15–20 and 12–14 percentage points, respectively. A longer time since HSCT was associated with lower donor DNA levels in nail samples, whereas donor–recipient sex matching correlated with higher admixture (~7–8 percentage points). These associations likely reflect a combination of biological and measurement-related factors. Since Y-chromosomal and amelogenin markers were excluded from donor fraction estimation, the observed sex-match effect is unlikely to be a simple assay artifact. Better HLA compatibility and related donors may reduce sustained trafficking of donor-derived leukocytes into epithelial compartments, while longer post-transplant intervals allow re-establishment of recipient-derived cells in some tissues. Sex-matched transplants may reduce assay-level discrimination (e.g., loss of Y-marker confirmation), potentially elevating apparent admixture. Regardless of mechanism, the magnitude of these effects is clinically meaningful and should inform both pre-test planning and post-test interpretation, particularly for borderline germline findings.

Our cross-sectional design does not permit causal inference, and we therefore avoid attributing these associations to specific biological mechanisms such as epithelial turnover rates or graft-cell homing kinetics. We also did not model platelet transfusion exposure or clearance, as our focus was on steady-state extra-hematopoietic chimerism rather than acute transfusion dynamics.

Taking together, these data define a practical framework for post-HSCT germline diagnostics. Eyebrow follicles minimize donor-derived DNA across assays, whereas buccal and nail samples frequently show admixture levels that can compromise germline inference. Although STR systematically reports higher donor-derived DNA-fractions than NGS at low admixture, both methods support the same clinical message: tissue choice matters, and eyebrow follicles are optimal for routine practice. Several limitations merit consideration. Eyebrow sampling was not performed in all patients due to its later introduction into the protocol or physician discretion (e.g., during chemotherapy), and DNA yield from eyebrow follicles is lower than from other tissues. Nevertheless, sufficient material for broader assays such as WES or WGS can be obtained if enough follicles are collected. While reduced NGS depth may limit sensitivity for detecting very low-frequency variants, our regression results were robust to adjustment for sequencing depth.

**Figure 2:**
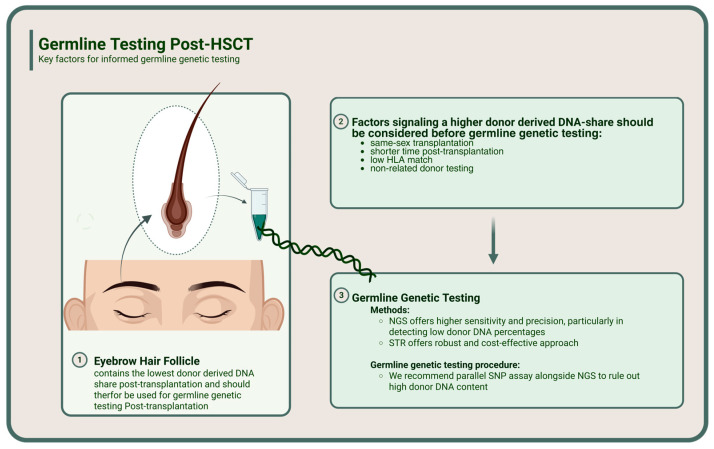
Protocol for Germline Testing Post-HSCT proposes a workflow for germline genetic testing after HSCT when pre-transplant peripheral blood is unavailable. Step 1: obtain eyebrow hair follicles as the default tissue (or nails if eyebrow sampling is not feasible) and perform a rapid SNP-based chimerism QC assay. Step 2: if donor-derived DNA fraction is <5%, proceed with germline testing; if 5–10%, proceed with caution, reviewing borderline heterozygous and CNV calls or consider recollection; if >10%, avoid germline inference from that specimen and seek an alternative tissue where possible. Figure created with BioRender.com. https://BioRender.com/n2bac3n (accessed on 27 December 2025).

Therefore, we recommend a two-step workflow for post-HSCT germline testing: (i) collect eyebrow follicles as the default tissue (or, if eyebrow sampling is not feasible, nails after a longer period post-HSCT), and (ii) perform a rapid SNP-based chimerism QC assay before comprehensive germline analysis (see Figure 2 and Table 3). 

From an implementation standpoint, eyebrow follicles were typically feasible and well tolerated when collected but may be less practical during intensive therapy or in patients with limited hair availability; in such cases, nails can serve as a secondary option provided that a rapid chimerism QC step is applied. Scalp hair follicles are a common alternative in some centers; however, published research has shown that hair follicles after HSCT can still contain donor-derived DNA, and we therefore recommend the same SNP-based QC approach irrespective of whether eyebrow or scalp hair follicles are used. Based on the observed donor fraction distributions (eyebrow NGS median 1%, maximum 4%, versus substantially higher levels in nails and buccal swabs), we propose preliminary QC thresholds for targeted SNP panels: donor-derived DNA fraction < 5% is generally compatible with routine germline interpretation; 5–10% constitutes a caution zone requiring careful review of borderline heterozygous calls and CNV or VAF-based QC metrics or consideration of an alternative tissue; and >10% should prompt avoidance of germline inference from that specimen. These thresholds are heuristic and specific to the depth and design of our 34-SNP panel; future studies should refine assay-specific cutoffs for ultra-deep panels or low-pass WGS, exome-, and genome-scale testing.

In early post-HSCT settings, and in sex-matched, unrelated, or sub optimally HLA-matched transplants, buccal and nail specimens should be avoided as first-line matrices and interpreted with caution when eyebrow samples are unavailable. Prospective, multicenter, and longitudinal studies are needed to calibrate assay-specific QC thresholds and to define the temporal dynamics of extra-hematopoietic chimerism. Nevertheless, the present data already support immediate implementation of an eyebrow-first plus SNP-QC strategy to improve the reliability of germline testing after HSCT.

## 4. Materials and Methods

### 4.1. Cohort

We analyzed 46 post-transplant recipients (33 pediatric; 13 adult). Forty-five received allogeneic HSCT; one autologous HSCT patient served as an internal control to verify near-zero background in non-chimeric tissues. This single autologous recipient was used only as an analytic control to confirm near-zero background chimerism and was not intended as a denominator for estimating effect sizes. Table 4 summarizes characteristics of the probands.

No participant had hematologic relapse during follow-up. Inclusion required prior HSCT and consent. Ethics approval was obtained (ID 186/21-ek); national registration DRKS00032352. Written consent/assent was obtained. Written informed consent was obtained from all individual participants (or their legal guardians) included in this study, in accordance with the Declaration of Helsinki and local institutional ethic committee requirements.

### 4.2. Material

We analyzed three materials: buccal swab, nail, and eyebrow hair samples. All these tissues offer a non-invasive and easily accessible DNA source. We collected the tissue samples during the transplant follow-up. To attribute individual gene sequences to either the donor or recipient, we established genetic profiles using donor DNA reference samples from pre-transplantation blood samples. Pre-transplantation blood was chosen as reference standard because it unequivocally represents the recipient’s germline DNA before donor-derived chimerism. The buccal swab samples were obtained using the ORAcollect-DNA kit (DNA Genotek Inc., Stittsville, ON, Canada), following the manufacturer’s protocol. Participants were instructed to refrain from eating or drinking for 30 minutes prior to sampling. The swab was rubbed multiple times along the inner cheek (between the lower jaw and buccal mucosa), consistent with the kit’s anatomical guidance. A demonstration video was made available to families (Video available online: https://www.youtube.com/watch?v=5VQI4GYwFLU, accessed on 19 November 2025) [19]. However, buccal swab collection was not systematically timed with respect to active oral mucositis or other inflammatory episodes; therefore, the influence of oral inflammation on donor-derived DNA fraction could not be evaluated in this study. To ensure an adequate amount of DNA in our nail samples, we required at least five fingernail or toenail samples collected after a 2-week period without cutting nails. Nail samples were cleaned prior to DNA extraction using a standardized protocol involving thorough washing with soap and water followed by ethanol wiping to reduce surface contamination. For eyebrow hairs, we required 15 hairs with hair roots. Eyebrow follicles were added to the protocol at a later stage and not requested in all patients, particularly when treating physicians advised against it (e.g., during chemotherapy), but when collected, they proved technically robust and well tolerated. The buccal swab, nails, and eyebrow hairs were stored at room temperature until DNA isolation. Not all patients provided all three tissue types. Missing eyebrow samples reflect participant’s choice. No additional exclusion was applied at the patient level.

### 4.3. Methods

#### 4.3.1. Sample Collection and DNA Extraction

We extracted DNA from buccal swabs using the MagCore Genomic DNA Whole Blood Kit 101 and the MagCore^®^ HF 16 Plus II instrument (RBC Bioscience, New Taipei City, Taiwan). For nail DNA extraction, we used the DNeasy Blood & Tissue Kit (Qiagen, Hilden, Nordrhein-Westfalen, Germany). For eyebrow DNA extraction, we utilized the innuPREP Forensic Kit (Analytic Jena, Jena, Germany). Furthermore, we used DNA from blood samples collected before transplantation as reference materials for donors and recipients. We measured DNA concentration using the Tecan Plate Reader Infinite^®^ 200 PRO (Tecan, Männedorf, Switzerland). In cases of low DNA concentrations, we employed NanoDrop 2000 (Thermo Fisher Scientific, Waltham, MA, USA) or Qubit 4.0 (Thermo Fisher Scientific, Waltham, MA, USA). DNA yield varied by tissue type, with median concentrations of 23.4 ng/µL from nail, 15.8 ng/µL from buccal swabs, and 7.9 ng/µL from eyebrow follicles (Table 4). For **NGS**, a minimum **DNA input of 10 ng** was required, while the **STR** protocol required pre-diluted **DNA at 40 ng/µL**. Due to variability in DNA yield—particularly in eyebrow follicles—libraries could not always be normalized to identical molar input and were pooled by fixed volume, contributing to lower sequencing depth in eyebrow samples. To mitigate the impact of depth variability, we applied prespecified marker-level QC thresholds (coverage and allelic balance) and summarized donor fraction as the median across informative loci.

#### 4.3.2. Next-Generation Sequencing

For NGS, we used the commercially available EasySeq™ NGS Reverse Complement PCR Human Exome Sample Tracking Kit (NimaGen, Nijmegen, The Netherlands), targeting 34 SNP loci, allowing us to discern and quantify the presence of donor-derived DNA in each tissue type (see Table 5). 

NGS analysis was conducted using either NextSeq 550 or NovaSeq 6000 from Illumina (San Diego, CA, USA). NGS sequence analysis was facilitated by the Varvis^®^ software Version 1.12 from Limbus (Rostock, Mecklenburg-Vorpommern, Germany). NGS non-informative markers were prespecified as (i) insufficient coverage; (ii) identical donor/recipient base; (iii) allelic fractions not summing to 100% with a ±3% tolerance (fractions rescaled if within tolerance); (iv) patterns indicating technical artifact or mosaic mixture (predefined intermediate allele fraction bands). Per patient tissue assay, the donor DNA fraction was the median across informative markers.

#### 4.3.3. Short Tandem Repeat (STR) Assay

We developed an in-house 27-marker STR multiplex based on previously published marker sets (see Table 6).

PCR used the Multiplex PCR Kit (Qiagen) with input 40 ng/µl (pre-diluted). Amplicons were separated on an Applied Biosystems™ 3500 Genetic Analyzer (Life Technologies Corporation, Pleasanton, CA, USA) and were analyzed in GeneMapper^®^ Software, version 5 (Life Technologies Corporation, Carlsbad, CA, USA). STR non-informative markers were defined as (i) insufficient signal; (ii) identical donor/recipient microsatellite profiles; (iii) discordant lengths inconsistent with donor/recipient references (e.g., spurious peaks). Amelogenin and other Y-chromosome markers were used to confirm sample assignment but excluded from chimerism estimates. The patient-tissue-assay donor DNA fraction was the median across informative loci. In calculating the donor-derived DNA fraction, our empirical analysis used the statistical software Stata, version 16 (StataCorp LLC, College Station, TX, USA). To minimize bias, wet-lab analyses were conducted blinded to clinical covariates and donor/recipient status; these data were linked only at the statistical analysis stage. We developed a custom program code to classify results from both STR and NGS analyses as either “informative” or “not informative” on a per-marker, per-patient basis. By systematically identifying uninformative patient–marker combinations, this approach minimized potential human reporting errors. We then computed donor-derived DNA fractions for each patient–marker combination based on the observed genetic marker data. 

For NGS analysis, a patient–marker combination was classified as *non-informative* under any of the following conditions: (i) insufficient sequencing coverage; (ii) identical base calls in donor and recipient reference DNA; (iii) allelic fractions not summing to 100%, allowing a tolerance of ±3% (fractions within this range were rescaled to 100%); or (iv) allele patterns consistent with technical artifact or mosaic mixture, defined as hybrid base combinations outside the expected donor or recipient genotypes. Markers were classified as *technical artifact or mosaic* when the fraction of a single base fell within intermediate ranges indicative of mixed signals (10–40% or 60–90%). To mitigate the impact of measurement noise around these thresholds, intermediate values were discretized to 0%, 50%, or 100% before downstream analysis.

For STR analysis, a patient–marker combination was classified as non-informative under any of the following conditions: (i) insufficient signal intensity resulting in missing or unreliable allele calls; (ii) identical microsatellite profiles in donor and recipient reference samples; or (iii) discordance between the expected donor or recipient allele lengths and the observed STR peaks in post-transplant samples, consistent with technical artifacts or mosaic mixtures.

**Figure 3:**
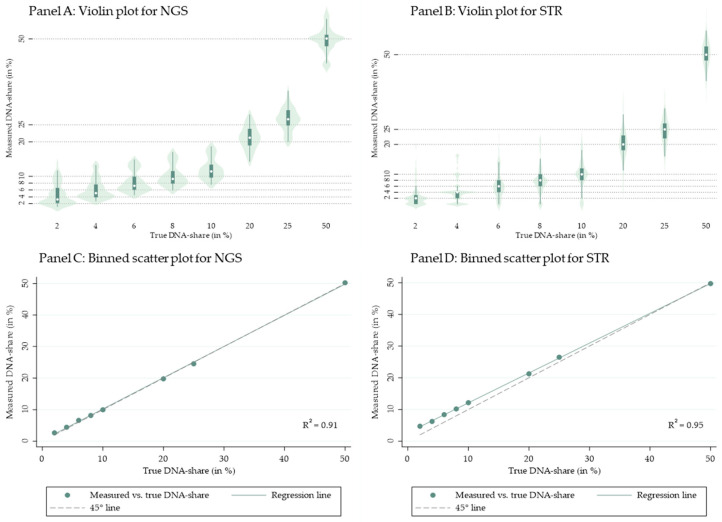
Dilution series benchmarking of donor-derived DNA fraction estimation by NGS and STR: illustrates the results of the dilution series using NGS and STR, applying our code. Panel A (B) displays violin plots for NGS (STR). Boxplots represent the distribution of DNA proportions within each dilution across all markers. Median DNA proportions are marked by a white dot. Panel C (D) displays binned scatter plots and a regression line from regressing measured on true DNA shares against a 45-degree line. Across all panels, the x-axis shows the actual DNA proportions of the eight diluted samples ranging from 2% to 50%; the y-axis displays the measured DNA proportions based on our analysis.

To ensure consistency across assays, Y-chromosomal and amelogenin markers were excluded from donor fraction estimation in both NGS and STR analyses. These markers were retained solely for sample identity confirmation. Across the dataset, we evaluated 3689 patient–marker combinations for NGS and 3120 for STR; of these, 2140 (NGS) and 1680 (STR) were classified as informative based on prespecified criteria. The average number of informative markers per tissue was 18 for buccal swabs, nails, and eyebrow follicles in the NGS assay, and 17, 13, and 12, respectively, in the STR assay. To mitigate the influence of outliers, donor-derived DNA fractions were summarized as patient-specific medians across informative markers for each tissue and method. To benchmark analytic performance, we generated eight DNA mixtures spanning 2–50% donor DNA from two non-transplanted control samples and analyzed them using both NGS and STR (Figure 3).

Both methods demonstrated high linearity between expected and measured donor fractions; the distributions by mixture level and median deviations are shown in Figure 3. Measured and true DNA-shares closely align, supporting the validity of our method.

### 4.4. Empirical Analysis

#### 4.4.1. Data Collection and Overview

Table 4 summarizes the final dataset comprising 45 patients and reports descriptive statistics for donor-derived DNA fractions, DNA input concentrations for both next-generation sequencing (NGS) and short tandem repeat (STR) analyses, and key NGS quality metrics, including total reads and average sequencing depth by tissue. These parameters are relevant for quantitative interpretation because sequencing depth and input DNA can influence the sensitivity of donor fraction estimates.

Among tissues, nail samples exhibited the highest sequencing yield, with a mean of 180,013 total reads and an average depth of 1507 per sample. In contrast, eyebrow follicle samples showed lower sequencing output, with a mean of 70,578 reads and an average depth of 639. Table 4 also reports cohort characteristics that were incorporated as control variables in the regression analyses (Our statistical analysis below will also control the average depth and total reads). At a median eyebrow depth of 639, a 5% donor fraction at a heterozygous locus corresponds to approximately 30–35 supporting reads. This depth is sufficient for the intended targeted 34-SNP quality control panel but provides limited sensitivity for detecting very low-frequency (<2–5%) donor-derived or mosaic variants, consistent with the dilution series results shown in Figure 1. The single autologous transplant case was excluded from all regression analyses due to the absence of informative markers and is not included in the dataset summarized in Table 4. 

#### 4.4.2. Regression Analysis

To investigate the statistical variations in donor-derived DNA among different tissue types, we use linear regression analysis. We estimate the following equation using ordinary least squares (OLS):

(1) $$y_{i}=\beta_{0}+\beta_{1}{D\text{\_}STR}_{i}+\beta_{2}{D\text{\_}BS}_{i}+\beta_{3}{D\text{\_}Na}_{i}+\phi_{i}^{\prime }\gamma +\varepsilon_{i},$$

where $y_{i}$ denotes the donor-derived DNA fraction of patient i. ${D\text{\_}STR}_{i}$, ${D\text{\_}BS}_{i}$, ${D\text{\_}Na}_{i}$ are dummies for data points from STR, buccal swab tissues, and nail tissues. The omitted categories are NGS and eyebrow hair follicle. The coefficient $\beta_{1}$, therefore, indicates the difference in donor-derived DNA fraction between STR and NGS analyses, whereas $\beta_{2}$ and $\beta_{3}$ measure the difference in donor-derived DNA fractions between buccal swab and eyebrow hair follicle tissues and between nail and eyebrow hair follicle tissues, respectively. $\beta_{0}$ captures the intercept and the vector $\phi_{i}^{\prime }$ contains control variables with coefficient values γ (to be discussed below). The term $\varepsilon_{i}$ represents the statistical error term. Since individuals may enter multiple times with multiple tissue samples into the regression analysis, we cluster standard errors at the patient level.

## Figures and Tables

**Table 1:** Differences in donor-derived DNA fraction across tissues presents summary statistics for donor-derived DNA fractions for each tissue. Columns 1, 2, 3, 4, 5, and 6 report means, medians, standard deviations maxima, minima, and the number of individuals with non-missing information. The data were self-collected as described in Materials and Methods.

Differences in donor-derived DNA fraction across tissues						
	Mean	Median	SD	Max	Min	N
Variable	(1)	(2)	(3)	(4)	(5)	(6)
Median donor fraction per nail sample (NGS)	0.19	0.08	0.24	0.89	0.00	45
Median donor fraction per nail sample (STR)	0.29	0.16	0.28	0.94	0.01	44
Median donor fraction per buccal swab sample (NGS)	0.25	0.20	0.18	0.87	0.00	45
Median donor fraction per buccal swab sample (STR)	0.29	0.24	0.19	0.88	0.03	45
Median donor fraction per eyebrow follicle sample (NGS)	0.01	0.01	0.01	0.04	0.00	28
Median donor fraction per eyebrow follicle sample (STR)	0.03	0.03	0.03	0.13	0.00	28

**Table 2:** OLS estimates of Equation (1). Columns 1–5 pool NGS and STR observations; columns 6–7 report method-specific regressions (STR and NGS). Standard errors are clustered at the patient level (in parentheses). Significance: * *p* < 0.10, ** *p* < 0.05, *** *p* < 0.01.

Regression analysis							
	*Dep. Variable: Median donor-derived DNA fraction*						
	NGS & STR (1)	NGS & STR (2)	NGS & STR (3)	NGS & STR (4)	NGS & STR (5)	STR (6)	NGS (7)
STR (1/0)	0.061 *** (0.007)	0.062 *** (0.007)	0.062 *** (0.007)	0.059 *** (0.007)	0.060 *** (0.007)		
Buccal swab (1/0)		0.251 *** (0.027)	0.252 *** (0.036)	0.227 *** (0.032)	0.289 ** (0.111)	0.240 *** (0.036)	0.225 *** (0.035)
Nail (1/0)		0.220 *** (0.038)	0.221 *** (0.038)	0.220 *** (0.040)	0.491 *** (0.147)	0.261 *** (0.047)	0.186 *** (0.037)
Buccal swab (1/0) × HLA match 10 (1/0)					−0.053 (0.117)		
Nail (1/0) × HLA match 10 (1/0)					−0.196 (0.136)		
Buccal swab (1/0) × Log (days after transplantation)					−0.003 (0.028)		
Nail (1/0) × Log (days after transplantation)					−0.110 ** (0.044)		
Log (DNA concentration)				−0.006 (0.024)	−0.017 (0.024)	−0.006 (0.028)	0.002 (0.023)
Same sex (1/0)				0.078 *** (0.026)	0.078 *** (0.024)	0.082 *** (0.030)	0.071 ** (0.027)
Log (age at transplantation)				0.031 * (0.017)	0.026 (0.016)	0.035 * (0.019)	0.027 (0.017)
Log (days after transplantation)				−0.042 ** (0.018)	0.003 (0.019)	−0.042 ** (0.019)	−0.035 * (0.019)
Bone marrow transplant (1/0)				0.040 (0.039)	0.035 (0.038)	0.053 (0.043)	0.018 (0.042)
Complication after transplantation (1/0)				−0.008 (0.034)	−0.004 (0.033)	−0.003 (0.039)	−0.009 (0.033)
Blood group compatibility (1/0)				0.026 (0.031)	0.021 (0.031)	0.031 (0.035)	0.021 (0.036)
HLA match 10 (1/0)				−0.186 *** (0.068)	−0.070 ** (0.032)	−0.196 *** (0.072)	−0.153 * (0.082)
Graft vs. Host Disease (1/0)				0.022 (0.036)	0.022 (0.035)	0.023 (0.042)	0.017 (0.033)
Matched rhesus factor (1/0)				0.032 (0.036)	0.035 (0.034)	0.034 (0.041)	0.030 (0.034)
Matched related donor (1/0)				−0.121 *** (0.036)	−0.120 *** (0.037)	−0.136 *** (0.042)	−0.106 *** (0.035)
Log (reads)							−0.029 (0.020)
Log (depth)							0.017 (0.015)
Regression constant	0.171 *** (0.018)	−0.009 ** (0.004)	−0.009 (0.024)	0.043 (0.096)	0.038 (0.084)	0.070 (0.105)	0.246 * (0.143)
Patient fixed effects	no	no	yes	no	no	no	no
Observations	235	235	235	217	217	108	109
R-squared	0.019	0.227	0.581	0.374	0.431	0.381	0.362

**Table 3:** Comparison of non-blood tissues for post-HSCT Germline Testing in this Study: Eyebrow follicles yielded the lowest donor-derived DNA fractions across both NGS and STR methods, making them the preferred matrix when pre-HSCT blood is unavailable. Nails offer a viable alternative with moderate donor admixture and good DNA yield. Buccal swabs, despite ease of collection, showed the highest admixture and should be avoided unless an SNP-based QC confirms low donor DNA levels.

comparison of non-blood tissues for post-HSCT germline testing in this study						
*Tissue*	*Collection*	*DNA yield*	*Donor %* *(NGS/STR)*	*Pro*	*Con*	*Use*
		*median*				
**Eyebrow** **follicles**	15 hairs including roots	7.9 ng/µL	0.01/0.03	Lowest Admixture, non-invasive	May not be feasible during chemotherapy	**Default** + SNP-based QC if borderline
**Nail**	≥5 nails after 2-week growth	23.4 ng/µL	0.08/0.16	Non-invasive; good yield; stable sample	Moderate Donor- admixture	**Backup** if eyebrows are unavailable + mandatory QC
**Buccal** **swab**	no food/drink 30 min prior	15.8 ng/µL	0.20/0.24	Easy, high yield, widely used	Highest Donor admixture	**Avoid** post-HSCT unless QC confirms low donor fraction

**Table 4:** Summary Statistics of our data. Columns 1, 2, 3, 4, 5, and 6 report means, medians, standard deviations, maxima, minima, and the number of individuals with non-missing information. For NGS, a minimum DNA input of 10 ng was required, while the STR protocol required pre-diluted DNA at 40 ng/µL. Lower yields from eyebrow samples partly explain the reduced sequencing depth observed in NGS for this tissue. The data were self-collected as described in Materials and Methods.

Summary statistics						
	Mean	Median	SD	Max	Min	N
Variable	(1)	(2)	(3)	(4)	(5)	(6)
DNA concentration, nail sample (ng/μL)	39.37	23.40	43.92	206.60	1.90	45
DNA concentration, buccal swab sample (ng/μL)	21.20	15.80	19.41	92.50	2.80	45
DNA concentration, eyebrow follicle sample (ng/μL)	10.62	7.90	7.99	29.50	2.80	28
Total reads per nail sample (NGS)	180,013	91,185	347,303	2,215,796	1935	45
Average depth per nail sample (NGS)	1507	762	2851	17,270	1	45
Total reads per buccal swab sample (NGS)	569,727	101,578	1,482,128	6,446,105	6148	45
Average depth per buccal swab sample (NGS)	5466	816	14,862	64,418	104	45
Total reads per eyebrow follicle sample (NGS)	70,578	70,739	55,794	195,410	3853	28
Average depth per eyebrow follicle sample (NGS)	639	607	466	1634	35	28
Age at transplantation (years)	18.77	8.00	22.30	72	0.40	45
Days after transplantation	1319	965	1319	6339	97	45
Gender donor (1 = male/0 = female)	0.78	1.00	0.42	1.00	0.00	45
Gender recipient (1 = male /0 = female)	0.58	1.00	0.50	1.00	0.00	45
Transplant type (1 = bone marrow/ 0 = periph. stem cell)	0.51	1.00	0.51	1.00	0.00	43
Complication after transplantation (1/0)	0.53	0.00	0.49	1.00	0.00	43
Graft vs. Host Disease (1/0)	0.60	1.00	0.49	1.00	0.00	43
HLA match (scale from 1 to 10, 10 being best)	9.9	10.00	0.29	10.00	8.00	44
Blood group compatibility (1/0)	0.49	0.00	0.51	1.00	0.00	43
Matched related donor (1/0)	0.24	0.00	0.43	1.00	0.00	45
Rhesus factor match (1/0)	0.73	1.00	0.45	1.00	0.00	45

**Table 5:** Genetic markers analyzed in NGS panels presents the genetic markers analyzed in the Next-Generation Sequencing (NGS) assay. NGS markers are single nucleotide polymorphisms (SNPs) with their corresponding rs-numbers. The genomic position indicates the specific location of each marker on the human genome (hg 38). Data from gnomAD v3.1.2 provides information on allele frequencies and mostly observed base in diverse human populations. AF = allele frequency.

Genetic Markers Analyzed in NGS Panels					
NGS Marker	Position	rs-Number	Chromosome	Reference	AF
rs9962023	23833905	rs9962023	18	T	0.6443
rs9532292	38859469	rs9532292	13	A	0.3905
rs760482	38782696	rs760482	22	A	0.3273
rs7465584	123975238	rs7465584	8	T	0.4508
rs7300444	884764	rs7300444	12	C	0.3992
rs6568050	112454808	rs6568050	X	T	0.5319
rs5930933	136349199	rs5930933	X	C	0.4882
rs577993	76706955	rs577993	9	C	0.5659
rs495680	33129519	rs495680	13	T	0.5482
rs4870723	120216440	rs4870723	8	A	0.5173
rs4735258	93923709	rs4735258	8	T	0.4354
rs4688963	5748177	rs4688963	4	T	0.4118
rs4617548	16111867	rs4617548	11	A	0.5201
rs4577050	34236747	rs4577050	15	G	0.6110
rs4148973	42903480	rs4148973	21	T	0.6049
rs3826616	63987229	rs3826616	18	A	0.5344
rs309557	83538811	rs309557	5	T	0.5006
rs2819561	4362083	rs2819561	3	A	0.6141
rs2296241	54169680	rs2296241	20	G	0.5059
rs2229546	67395837	rs2229546	1	C	0.6144
rs2159132	14102122	rs2159132	17	G	0.5331
rs2073787	110451457	rs2073787	X	T	0.4811
rs1805034	62360008	rs1805034	18	C	0.5634
rs1572983	101371346	rs1572983	9	C	0.6298
rs1536928	122629130	rs1536928	9	A	0.4642
rs1410592	179551371	rs1410592	1	G	0.6140
rs1381532	97428498	rs1381532	9	A	0.5314
rs1292053	59886176	rs1292053	17	A	0.4631
rs11158685	67575857	rs11158685	14	A	0.4831
rs10883099	98459557	rs10883099	10	G	0.52665
rs10373	6119441	rs10373	20	A	0.5209
rs1037256	73201609	rs1037256	17	G	0.5162
rs1026128	73200670	rs1026128	17	A	0.5139
rs10203363	227032260	rs10203363	2	C	0.4668

**Table 6:** Genetic markers analyzed in STR panels presents the genetic markers analyzed in the Short Tandem Repeat (STR) assay. STR markers are short tandem repeat regions. The genomic position indicates the specific location of each marker on the human genome (hg 38).

Genetic Markers Analyzed in STR Panels				
STR Marker				
Name and Genomic Position		Forward 5′–3′	Reverse 5′–3′	Dye
vWa	12p13.31	ccctagtggatgataagaataatcagtat	ggacagatgataaatacataggatggat	FAM™
SRY	Yp11.31	gaatattcccgctctccgga	gctggtgctccattcttgag	ROX™
FGA	4q28	gccccataggttttgaactca	tgatttgtctgtaattgccagc	FAM™
DXS981	Xq13.1	ctccttgtggccttccttaaatg	ttctctccacttttcagagtca	ROX™
DXS7133	Xq22.3	gcttccttagatggcattca	cttccaagaatcagaagtctcc	ROX™
D8S1179	8q24.13	tttttgtatttcatgtgtacattcg	cgtagctataattagttcattttca	Atto550
D7S820	7q21.11	tgtcatagtttagaacgaactaacg	ctgaggtatcaaaaactcagagg	Atto565
D5S818	5q23.2	gggtgattttcctctttggt	tgattccaatcatagccaca	HEX™
D3S1358	3p21.31	actgcagtccaatctgggt	atgaaatcaacagaggcttg	Atto565
D21S1439	21q22.13	cttctccgcctgaaaatgta	attagggtgtgggtcctagc	ROX™
D21S1435	21q21.3	ccctctcaattgtttgtctacc	acaaaaggaaagcaagagatttca	FAM™
D21S1412	21q22.2	cggaggttgcagtgagttg	gggaaggctatggaggaga	ROX™
D21S1411	21q22.3	ataggtagatacataaatatgatga	tattaatgtgtgtccttccaggc	Atto550
D21S1270	21q22.11	ctatcccactgtattattcagggc	tgagtctccaggttgcaggtgaca	FAM™
D21S11	21q21.1	tttctcagtctccataaatatgtg	gatgttgtattagtcaatgttctc	FAM™
D18S978	18q12.1	gtagatcttgggacttgtcaga	gtctcccatggtcacaatgct	Atto550
D18S535	18q12.3	cagcaaacttcatgtgacaaaagc	caatggtaacctactatttacgtc	FAM™

## Data Availability

The original contributions presented in this study are included in the article. Further inquiries can be directed to the corresponding authors.
